# NMI Regulates Adipose Adaptive Thermogenesis Through TLR4/IRF3 Signaling to Promote Obesity

**DOI:** 10.1002/advs.75921

**Published:** 2026-06-02

**Authors:** Ting‐Ting Li, Xin‐Yuan Zhao, Min Zhang, Zhuang‐Feng Weng, Xiao‐Ran Guo, Ying‐Fang Liu, Qiao‐Ping Wang, Huan‐Huan Liang

**Affiliations:** ^1^ Zhongshan School of Medicine, Sun Yat‐sen University Shenzhen Campus Sun Yat‐sen University Shenzhen China; ^2^ Shenzhen Key Laboratory for Systems Medicine in Inflammatory Disease Shenzhen China; ^3^ School of Pharmaceutical Sciences (Shenzhen) Sun Yat‐sen University Shenzhen China; ^4^ Laboratory of Metabolism and Aging School of Pharmaceutical Sciences (Shenzhen) Shenzhen Campus of Sun Yat‐Sen University Shenzhen China; ^5^ Department of Pharmacy the Third Affiliated Hospital of Sun Yat‐Sen University Guangzhou China; ^6^ Guangdong Provincial Key Laboratory of Diabetology the Third Affiliated Hospital of Sun Yat‐Sen University Guangzhou China

**Keywords:** N‐Myc and STAT interactor (NMI), adaptive thermogenesis, immunometabolism, PPAR, IRF3

## Abstract

Obesity‐associated inflammation is partly driven by damage‐associated molecular patterns (DAMPs). However, whether and how these molecules directly restrain energy expenditure and thereby exacerbate metabolic dysfunction remains unclear. Here, we identify N‐Myc and STAT interactor (NMI) as a stress‐responsive adipokine that suppresses adaptive thermogenesis. NMI expression and secretion are increased in adipocytes exposed to dietary stress and inflammatory cues. Genetic ablation of *Nmi* protects mice from diet‐induced obesity (DIO) through enhanced energy expenditure and cold tolerance driven by augmented brown adipose tissue (BAT) thermogenesis and white adipose tissue (WAT) browning. Mechanistically, adipose tissue‐derived NMI activates TLR4/IRF3 signaling, which transcriptionally represses the core thermogenic regulators PPARα, PGC‐1α, and UCP1. Therapeutically, neutralization of NMI with a monoclonal antibody ameliorates obesity and reduces adipose tissue macrophage infiltration in DIO mice. Together, our findings establish NMI as an adipokine that couples inflammatory signaling with suppressed energy expenditure, highlighting its therapeutic potential for obesity and associated metabolic disorders.

## Graphical abstract

Adipose tissue‐derived NMI is secreted under metabolic stress and suppresses adaptive thermogenesis through TLR4/IRF3 signaling, repressing the PPARα/PGC‐1α/UCP1 thermogenic transcriptional program. Genetic ablation or anti ‐ NMI monoclonal antibody treatment enhances energy expenditure, protects against DIO, and ameliorates adipose tissue inflammation, positioning NMI as an immunometabolic regulator and potential therapeutic target for obesity.

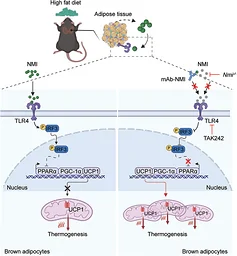

## 1 Introduction

Obesity is a major global health challenge [1, 2]. It primarily arises from a chronic energy imbalance, wherein caloric intake exceeds energy expenditure [3]. Enhancing energy expenditure through adaptive thermogenesis, defined as regulated heat production in response to environmental or physiological stimuli such as cold exposure or overfeeding, therefore represents a compelling therapeutic strategy, as it directly counteracts the positive energy balance that drives weight gain [4]. This process primarily occurs in brown and beige adipose tissues, which are characterized by the expression of uncoupling protein 1 (UCP1) to dissipate chemical energy as heat via mitochondrial uncoupling [5]. Upon cold stimulation, the β‐adrenergic receptor signaling cascade promotes the activation of the key transcriptional regulators of adaptive thermogenesis, including peroxisome proliferator‐activated receptor α (PPARα) and PPARγ coactivator 1α (PGC‐1α), which drive UCP1 expression and mitochondrial biogenesis, thereby maintaining body temperature and contributing to energy balance [4, 6, 7, 8, 9, 10].

Obesity and its associated metabolic complications are closely associated with chronic, low‐grade inflammation within adipose tissue [11], which exacerbates metabolic dysfunction and represents a promising therapeutic target [12]. Intriguingly, PPARs also suppress inflammation by antagonizing pro‐inflammatory pathways, including nuclear factor kappa‐B (NF‐κB), activator protein 1 (AP‐1), and signal transducer and activator of transcription (STAT) [13, 14]. This dual functionality in promoting energy expenditure and attenuating inflammation positions PPAR activation as a potentially synergistic strategy to ameliorate obesity and its sequelae. However, the molecular mechanisms that couple pro‐inflammatory signaling to the suppression of adipose adaptive thermogenesis remain poorly defined.

Damage‐associated molecular patterns (DAMPs) are endogenous molecules released under cellular stress and serve as critical mediators of sterile inflammation in obesity [15]. Typically lacking a classical signal peptide, DAMPs can be secreted from living cells via non‐canonical secretory pathways, vesicular transport, or membrane channels [16, 17, 18]. Extracellular DAMPs sustain chronic inflammation by activating pattern recognition receptors (PRRs) such as Toll‐like receptors (TLRs), thereby making DAMPs potential diagnostic biomarkers and therapeutic targets [19, 20]. Notably, in adipose tissue, TLR4 activation triggers the nuclear translocation of interferon regulatory factor 3 (IRF3) [21], an event that directly suppresses adaptive thermogenesis and promotes weight gain [22, 23]. These findings support the TLR4/IRF3 axis as a molecular link between inflammation and energy expenditure. Nonetheless, the upstream endogenous DAMPs that initiate this suppressive pathway remain unidentified.

N‐Myc and STAT interactor (NMI) is an interferon‐inducible protein with emerging roles in metabolism and inflammation. Beyond its established functions in immunity and tumorigenesis [24], NMI interacts with a key metabolic stress sensor. In pancreatic β‐cells, NMI binds to and modulates inositol‐requiring enzyme 1α (IRE1α) [25], a central hub in glucose and lipid metabolism as well as inflammation [26, 27, 28]. Notably, in adipocytes, IRE1α suppresses adaptive thermogenesis by repressing PGC‐1α [29]. Moreover, NMI has been strongly implicated in inflammatory processes and can function as a DAMP that sustains inflammation through TLR activation [19, 20, 30]. Given that NMI regulates IRE1α, a metabolic stress sensor [25, 26, 27, 28], and acts as a TLR4‐activating DAMP [30, 31], and that TLR4/IRF3 signaling suppresses adaptive thermogenesis [21, 22, 23], we hypothesize that NMI serves as a molecular integrator linking pro‐inflammatory signaling to the suppression of adaptive thermogenesis.

In this study, we demonstrate that *Nmi* deficiency protects mice from diet‐induced obesity (DIO) and metabolic dysfunction, as evidenced by increased energy expenditure, improved cold tolerance, and elevated thermogenic gene expression. Mechanistically, adipose tissue‐derived NMI activates TLR4/IRF3 signaling to suppress PPARα‐driven thermogenic transcription, thereby inhibiting UCP1‐mediated adaptive thermogenesis. Therapeutically, NMI neutralization via a monoclonal antibody ameliorates obesity and attenuates adipose tissue macrophage infiltration. Collectively, our findings establish NMI as a critical link between inflammatory and metabolic pathways, revealing its potential as a therapeutic target for obesity and associated metabolic disorders.

## 2 Results

### 2.1 *Nmi* Deficiency Protects Against DIO and Metabolic Dysfunction

**FIGURE 1:**
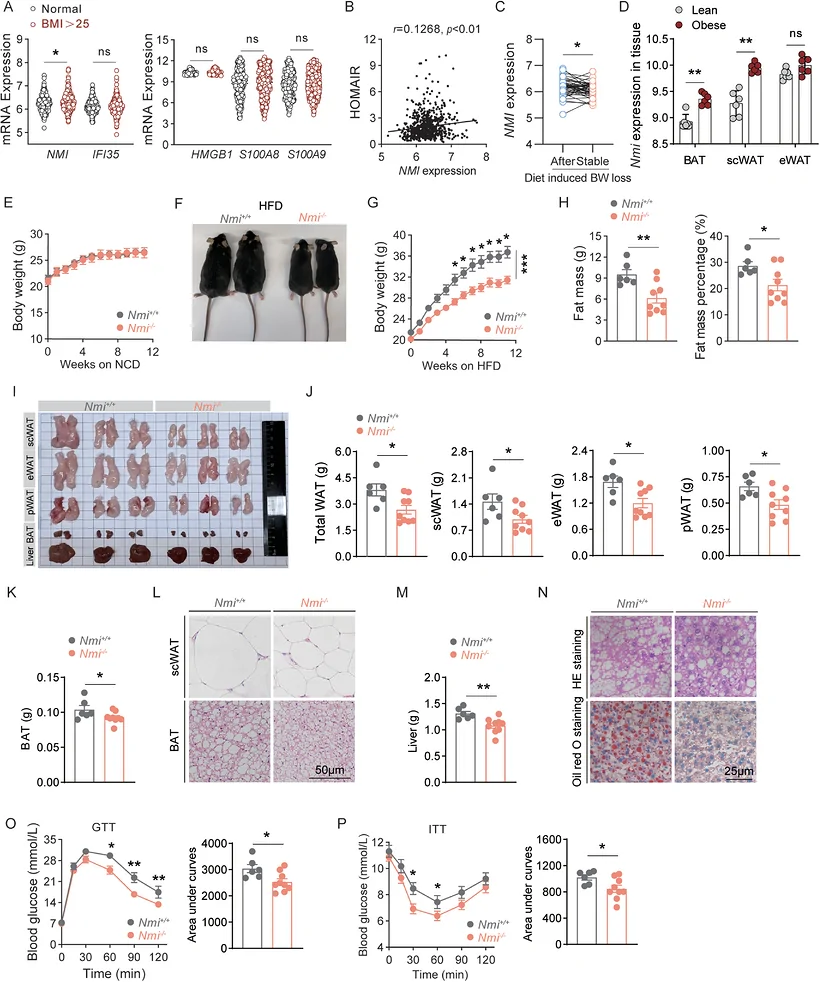
*Nmi* deficiency attenuates HFD‐induced adiposity and hepatic steatosis. (A) mRNA expression of damage‐associated molecular pattern (DAMP) genes in normal and overweight individuals (*n* = 770). (B) Pearson correlation between *NMI* expression and homeostatic model assessment of insulin resistance (HOMA‐IR) in humans (*n* = 770). (C) Changes in *NMI* expression in adipose tissue after dietary intervention‐induced weight loss and during the maintenance period in a weight‐loss cohort (*n* = 46). (D) *Nmi* mRNA expression in adipose tissues from lean and obese mice (*n* = 6). (E) Body weight (BW) of WT (*Nmi^+/+^*) and *Nmi*‐deficient (*Nmi^−/−^*) mice fed normal chow diet (NCD) (*n* = 9–12). (F, G) BW of *Nmi^+/+^* and *Nmi^−/−^* mice under high‐fat diet (HFD) for 12 weeks. Representative images (F) and BW growth curve (G, *n* = 6–9). (H) Fat mass and fat mass percentage measured by magnetic resonance imaging (MRI) in *Nmi^+/+^* and *Nmi^−/−^* mice after 12 weeks of HFD (*n* = 6–9). (I, J) Fat depot weights in *Nmi^+/+^* and *Nmi^−/−^* mice after 12 weeks of HFD. Representative images (I) and weights of total white adipose tissue (total WAT), subcutaneous WAT (scWAT), epididymal WAT (eWAT), and perirenal WAT (pWAT) (J, *n* = 6–9). (K) Brown adipose tissue (BAT) weight in *Nmi^+/+^* and *Nmi^−/−^* mice after 12 weeks of HFD (*n* = 6–9). (L) Representative images of H&E staining in scWAT and BAT after 12 weeks of HFD. (M) Liver weight after 12 weeks of HFD (*n* = 6–9). (N) Representative images of liver H&E and Oil Red O staining after 12 weeks of HFD. (O, P) Glucose tolerance test (GTT, O) and insulin tolerance test (ITT, P) after 12 weeks of HFD, quantified by area under the curve (AUC, *n* = 6–9). Data are represented as mean ± SEM. Statistical analyses: two‐tailed unpaired Student's t‐test (A, D, H, J, K, M, AUC of O, P), Pearson correlation analysis (B), two‐tailed paired Student's t‐test (C), two‐way ANOVA followed by Bonferroni's multiple comparisons test (E, G, O, P). *p < 0.05, **p < 0.01, ***p < 0.001; ns, not significant.

Analysis of human and mouse cohorts revealed an association between NMI expression and obesity. In overweight individuals, adipose *NMI* expression was upregulated and positively correlated with homeostatic model assessment of insulin resistance (HOMA‐IR) (Figure 1A,B). Consistently, *NMI* expression was significantly reduced in the weight‐loss intervention maintenance cohort (Figure 1C). *Nmi* was also upregulated in brown adipose tissue (BAT) and subcutaneous white adipose tissue (scWAT) of obese mice (Figure 1D, Figure S1A). To characterize *Nmi* expression across cell types, we analyzed public single‐nucleus RNA‐seq data and found *Nmi* was enriched in adipocytes and immune cells (Figure S1B). Notably, unlike typical DAMPs, *Nmi* expression was rapidly downregulated following both acute and chronic cold exposure (Figure S1C,D), suggesting a potential negative role in adaptive thermogenesis, rather than merely reflecting a passive stress response.

To investigate the functional role of NMI in adaptive thermogenesis, we generated global *Nmi* knockout (*Nmi^−/−^*) mice (Figure S2A,B). On a normal chow diet (NCD), *Nmi^−/−^* mice exhibited no difference in body weight (BW), adipose morphology, or glucose homeostasis compared to wild‐type (WT, *Nmi^+/+^*) littermates (Figure 1E, Figure S2C–F). However, when challenged with a high‐fat diet (HFD, 60% kcal from fat), *Nmi^−/−^* mice showed significant resistance to weight gain, with 19.4% lower BW than *Nmi^+/+^* littermates after 12 weeks (Figure 1F,G). Magnetic resonance imaging (MRI) analysis demonstrated a significant reduction in fat mass and no alteration in lean mass (Figure 1H, Figure S2G). Consistently, the weights of all major adipose depots, including scWAT, epididymal WAT (eWAT), perirenal WAT (pWAT), and BAT were significantly reduced in *Nmi^−/−^* mice (Figure 1I–K), which was accompanied by an increased proportion of smaller adipocytes (Figure 1L, Figure S2H).

HFD‐fed *Nmi^−/−^* mice also displayed several systemic metabolic improvements, including reduced liver weight and hepatic lipid accumulation (Figure 1M,N), as well as enhanced glucose tolerance and insulin sensitivity (Figure 1O,P). Collectively, these results demonstrate that NMI promotes the development of DIO and metabolic dysfunction, and global *Nmi* ablation confers substantial metabolic protection.

### 2.2 *Nmi* Deletion Counteracts Obesity by Enhancing Energy Expenditure

**FIGURE 2:**
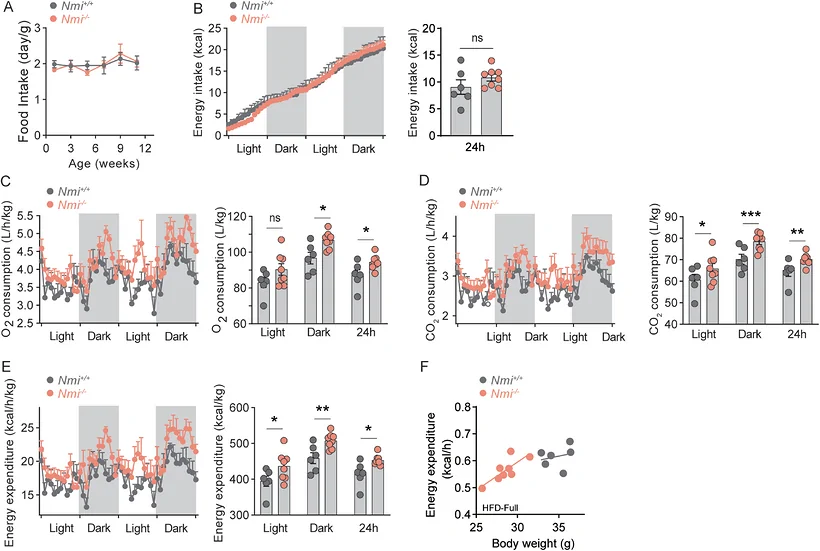
*Nmi* deficiency enhances energy expenditure. (A, B) Food consumption (A) and cumulative energy intake (B) in *Nmi^+/+^* and *Nmi^−/−^* mice (*n* = 6–8). (C‐E) Oxygen consumption (VO_2_, C), carbon dioxide production (VCO_2_, D) and total energy expenditure (E) in *Nmi^+/+^* and *Nmi^−/−^* mice (*n* = 6–8). (F) Analysis of covariance (ANCOVA) of energy expenditure with BW as a covariate (*n* = 6–8). Data are represented as mean ± SEM. Statistical analyses: two‐way ANOVA followed by Bonferroni's multiple comparisons test (A), two‐tailed unpaired Student's *t*‐test (B–E), ANCOVA with BW as covariate (F). *p < 0.05, **p < 0.01, ***p < 0.001; ns, not significant.

We next investigated whether the lean phenotype of HFD‐fed *Nmi^−/−^* mice stems from altered energy balance. Food intake and cumulative 48‐h energy intake in metabolic cages were comparable between *Nmi^−/−^* mice and wild‐type littermates (Figure 2A,B), ruling out reduced caloric intake as a contributing factor. Strikingly, *Nmi^−/−^* mice exhibited significantly increased whole‐body energy expenditure, as evidenced by elevated oxygen consumption (VO_2_) and carbon dioxide production (VCO_2_) (Figure 2C–E). This difference remained significant after normalization to BW (Figure 2F, Figure S3A), supporting the notion that the effect was not solely driven by differences in body mass. In contrast, the respiratory exchange ratio (RER) and physical activity were unchanged between genotypes (Figure S3B,C), indicating similar overall fuel oxidation patterns and locomotor activity. In addition, we preliminarily ruled out contributions from other factors, including altered nutrient absorption and fecal energy loss (Figure 1E, Figure S3D,E). Collectively, our data demonstrate that *Nmi* deletion protects against obesity primarily through enhanced energy expenditure.

### 2.3 *Nmi* Deficiency Enhances Thermogenic Capacity and Cold Tolerance

**FIGURE 3:**
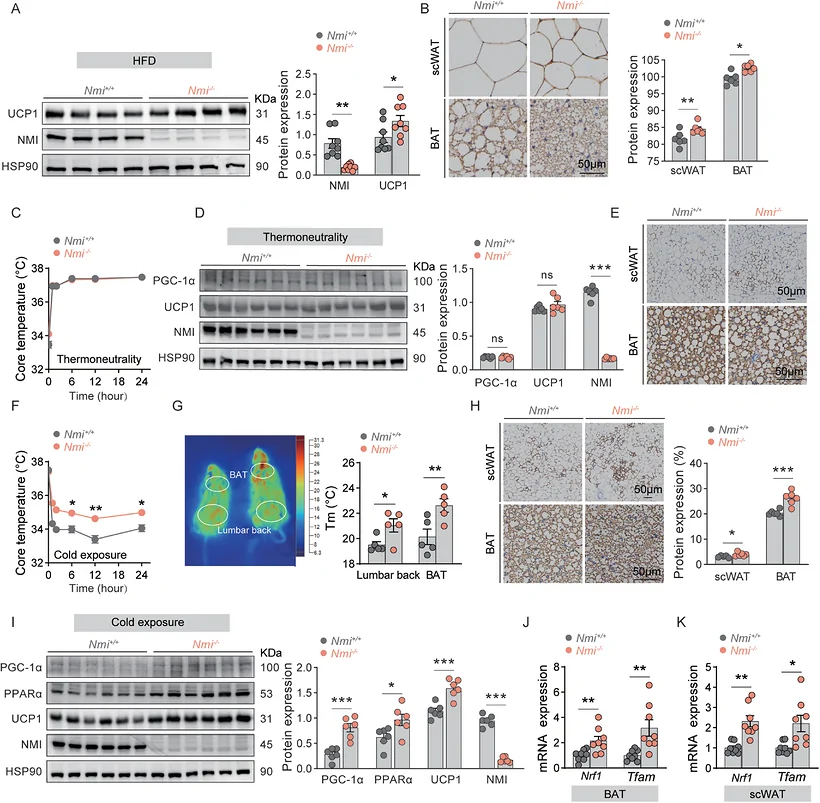
*Nmi* deficiency promotes BAT adaptive thermogenesis and scWAT browning in HFD‐fed mice. (A, B) UCP1 protein levels in adipose tissue of HFD‐fed *Nmi^+/+^* and *Nmi^−/−^* mice, assessed by Western blotting (A, *n* = 8) and immunohistochemistry (B, *n* = 6). (C) Time‐course changes in core body temperature over 24 h under thermoneutral conditions (30°C) in *Nmi^+/+^* and *Nmi^−/−^* mice (*n* = 12–14). (D) Thermogenic protein levels in BAT under thermoneutral conditions (30°C) in *Nmi^+/+^* and *Nmi^−/−^* mice, determined by Western blotting (*n* = 6). (E) Representative UCP1 immunohistochemistry images in scWAT and BAT under thermoneutral conditions (30°C). (F, G) Core body temperature (F, *n* = 5) and surface temperature (G, *n* = 7) in 8‐week‐old *Nmi^+/+^* and *Nmi^−/−^* mice under cold exposure (4°C), with representative infrared thermal images of the lumbar back region and interscapular BAT. (H) Representative UCP1 immunohistochemistry images in scWAT and BAT under cold exposure (4°C). (I) Thermogenic protein levels in BAT under cold exposure (4°C), determined by Western blotting (*n* = 6). (J, K) Mitochondrial biogenesis‐related gene expression in BAT (J) and scWAT (K) of *Nmi^+/+^* and *Nmi^−/−^* mice under HFD feeding (*n* = 8). Data are represented as mean ± SEM. Statistical analyses: two‐tailed unpaired Student's *t*‐test (A, B, D, G–I), two‐way ANOVA followed by Bonferroni's multiple comparisons test (C, F). *p < 0.05, **p < 0.01, ***p < 0.001; ns, not significant.

UCP1‐mediated adaptive thermogenesis in BAT and beige adipocytes is a central regulator of energy expenditure [6, 32, 33, 34]. Therefore, we investigated whether enhanced adaptive thermogenesis underlies the obesity resistance in *Nmi^−/−^* mice. Indeed, *Nmi* deletion significantly upregulated UCP1 protein levels in BAT of HFD‐fed mice (Figure 3A), with immunohistochemical staining confirming abundant UCP1 within multilocular adipocytes in both BAT and scWAT (Figure 3B).

We next assessed thermogenic capacity by challenging mice with cold exposure (4°C) [35, 36]. Under strict thermoneutral control conditions (30°C), core body temperature and thermogenic protein levels were comparable between the two genotypes (Figure 3C, Figure S3F), indicating no baseline differences in thermogenic regulators such as PGC‐1α and UCP1 (Figure 3D,E). However, upon cold exposure, *Nmi^−/−^* mice exhibited a significantly higher core body temperature throughout the 24‐h challenge (Figure 3F). Non‐invasive infrared thermography also revealed a similar phenotype, with significantly elevated surface temperatures over the intrascapular BAT and lumbar region (Figure 3G), consistent with heightened adaptive thermogenesis. This improved cold tolerance was accompanied by robust induction of thermogenic proteins in BAT and pronounced browning of scWAT (Figure 3H,I). Moreover, cold exposure triggered a more substantial reduction in adipocyte size in both scWAT and BAT of *Nmi^−/−^* mice compared to *Nmi^+/+^* littermates (Figure S3G,H).

Beyond UCP1 protein expression, we examined regulators of mitochondrial biogenesis, a key correlate of thermogenic capacity [37]. mRNA levels of key mitochondrial biogenesis regulators (*Nrf1, Tfam*) were elevated in both BAT and scWAT of HFD‐fed *Nmi^−/−^* mice (Figure 3J,K). Collectively, these findings demonstrate that *Nmi* deficiency enhances energy expenditure by promoting mitochondrial biogenesis, improving BAT function and promoting WAT browning, which together constitute a coordinated adaptive thermogenic response.

### 2.4 NMI Is Enriched in Mature Adipocytes and Upregulated in Obesity

**FIGURE 4:**
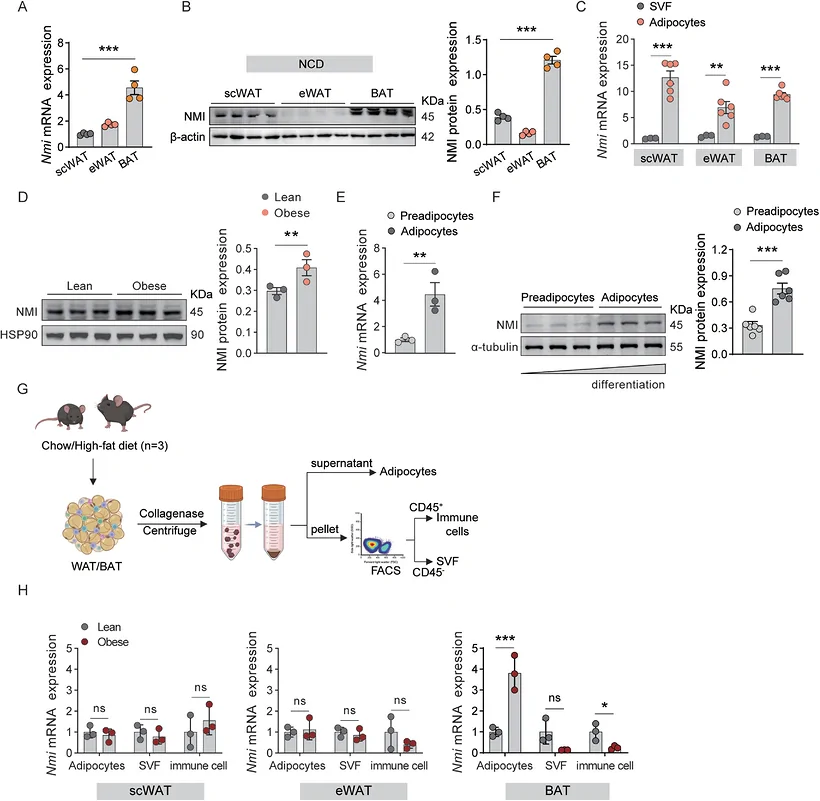
NMI is enriched in mature adipocytes. (A, B) *Nmi* mRNA expression (A) and NMI protein levels (B) across adipose depots in 8‐week‐old male mice (*n* = 4). (C) *Nmi* mRNA expression in stromal vascular fraction (SVF) and mature adipocytes from 8‐ to 10‐week‐old chow‐fed mice (*n* = 3–6). (D) NMI protein levels in BAT from lean and diet‐induced obesity (DIO) mice, determined by Western blotting (*n* = 3). (E, F) *Nmi* mRNA (E, *n* = 3) and NMI protein (F, *n* = 6) during 3T3‐L1 differentiation into beige adipocytes (Day 0 and Day 8). (G) Schematic of fluorescence‐activated cell sorting (FACS) strategy for separating adipocyte precursor cells (CD45^−^) and immune cells (CD45^+^) from the SVF. (H) *Nmi* mRNA levels in SVF, immune cells, and mature adipocytes from lean mice and DIO mice (*n* = 3). Data are represented as mean ± SEM. Statistical analyses: one‐way ANOVA followed by Bonferroni's multiple comparisons test (A, B), two‐tailed unpaired Student's t‐test (C‐F, H). *p < 0.05, **p < 0.01, ***p < 0.001; ns, not significant.

We first characterizedNMI expression across different adipose tissue depots in mice. NMI was ubiquitously expressed in scWAT, eWAT, and BAT, with both transcript and protein levels highest in BAT (Figure 4A,B). To identify its primary cellular source, we isolated the stromal vascular fraction (SVF) and mature adipocytes from NCD wild‐type mice. *Nmi* expression was enriched in mature adipocytes across all depots compared to the SVF (Figure 4C). Moreover, NMI abundance increased further in BAT under obese conditions (Figure 4D). This adipocyte‐enriched expression pattern was further corroborated by in vitro differentiation and browning of 3T3‐L1 preadipocytes, during which *Nmi*mRNA and NMI protein levels were significantly increased (Figure 4E,F).

To examine NMI levels under metabolic stress, we performed fluorescence‐activated cell sorting (FACS) on digested adipose tissue from both lean and obese mice to separate mature adipocytes, non‐immune stromal cells (CD45^−^) and immune cells (CD45^+^) (Figure 4G). Notably, *Nmi* expression was specifically upregulated in mature BAT adipocytes under obese conditions (Figure 4H). Together, these findings establish mature adipocytes, particularly those within BAT, as the primary source of NMI expression in adipose tissue. Its expression was further amplified by obesity and during in vitro adipocyte differentiation process, supporting a role for NMI in mediating thermogenic regulation in adipocytes.

### 2.5 RNA‐seq Reveals Enhanced PPAR Signaling in BAT of *Nmi^−/−^* Mice

**FIGURE 5:**
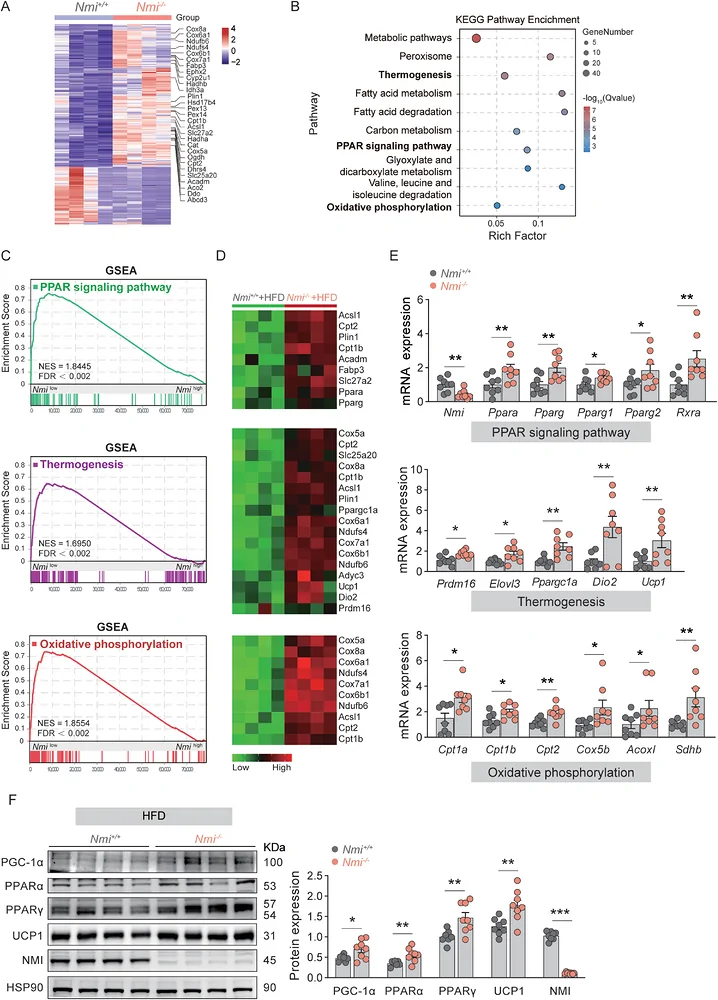
Transcriptomic analysis reveals enhanced PPAR signaling and adaptive thermogenesis in BAT of *Nmi^−/−^* mice. (A) RNA sequencing (RNA‐seq) of BAT from HFD‐fed *Nmi^+/+^* and *Nmi^−/−^* mice. Differentially expressed genes (DEGs, |log_2_ fold change|> 1, false discovery rate [FDR] < 0.05) were primarily enriched in adaptive thermogenesis and fatty acid oxidation pathways (*n* = 4). (B) Kyoto Encyclopedia of Genes and Genomes (KEGG) enrichment analysis of upregulated genes (|log_2_ fold change| > 1 and FDR < 0.05). (C) Gene set enrichment analysis (GSEA) showing major upregulated pathways, including PPAR signaling, adaptive thermogenesis, and oxidative phosphorylation (normalized enrichment score [NES] > 1.7 and FDR < 0.002). (D) Heatmap of pathway‐related genes (PPAR signaling, adaptive thermogenesis, and oxidative phosphorylation) from RNA‐seq (*n* = 4). (E, F) Validation of RNA‐seq results by quantitative PCR (E) and Western blotting (F) (*n* = 8). Data are represented as mean ± SEM. Statistical analyses for RNA‐seq: differential expression analysis was performed using DESeq2 (|log_2_ fold change| > 1, FDR < 0.05); KEGG and GSEA were performed using clusterProfiler. For validation experiments: two‐tailed unpaired Student's t‐test (E, F). *p < 0.05, **p < 0.01, ***p < 0.001; ns, not significant.

To elucidate the mechanism underlying NMI‐regulated adaptive thermogenesis, we performed RNA sequencing (RNA‐seq) on BAT samples from HFD‐fed *Nmi^−/−^* and wild‐type littermates. Kyoto Encyclopedia of Genes and Genomes (KEGG) pathway analysis revealed significant enrichment of pathways related to adaptive thermogenesis, PPAR signaling, and oxidative phosphorylation among upregulated genes in *Nmi^−/−^* mice (Figure 5A,B, Figure S4A). Gene Set Enrichment Analysis (GSEA) further confirmed positive enrichment of these pathways upon *Nmi* deletion (Figure 5C). Specifically, core genes within the PPAR axis, including genes involved in adaptive thermogenesis, fatty acid oxidation, and oxidative phosphorylation, were significantly upregulated (Figure 5D).

We next validated these findings by examining key pathway components using quantitative PCR (qPCR). Expression levels of PPAR family members, key thermogenic genes, and fatty acid oxidation markers were consistent with RNA‐seq data (Figure 5E). This transcriptional upregulation was further supported by increased protein levels of PGC‐1α, PPARα, PPARγ, and UCP1 (Figure 5F), suggesting that *Nmi* deficiency enhances these thermogenic pathways. Together, these data reveal that *Nmi* deficiency enhances the PPAR signaling pathway and its downstream thermogenic program in BAT.

### 2.6 NMI Represses Adaptive Thermogenesis by Suppressing PPAR Signaling

**FIGURE 6:**
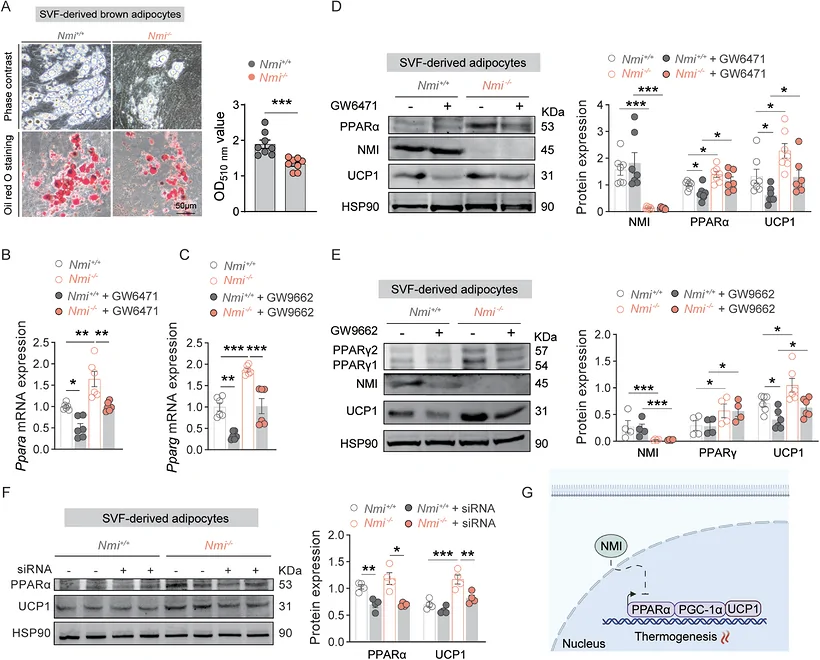
NMI regulates adaptive thermogenesis through PPAR signaling. (A) Triacylglycerol (TAG) content in differentiated primary brown adipocytes from *Nmi^+/+^* and *Nmi^−/−^* mice (*n* = 8). (B, C) Thermogenic gene expression in primary brown adipocytes treated with 10 µm GW6471 (PPARα antagonist), or 10 µm GW9662 (PPARγ antagonist) for 24 h (B) or 48 h (C) (*n* = 4–6). (D, E) Thermogenic protein levels in primary brown adipocytes treated as in (B, C) for 24 h (D) or 48 h (E) (*n* = 4–6). (F) Thermogenic gene expression in primary adipocytes transfected with scramble siRNA or *Ppara* siRNA (100 nm) for 7 days (*n* = 4). (G) Model of NMI‐mediated suppression of adaptive thermogenesis via PPAR signaling. NMI induces transrepression of PPARα and PPARγ transcriptional activity, leading to UCP1 downregulation (Created with BioRender.com). Data are represented as mean ± SEM. Statistical analyses: two‐tailed unpaired Student's t‐test (A), one‐way ANOVA followed by Bonferroni's multiple comparisons test (B–F). *p < 0.05, **p < 0.01, ***p < 0.001; ns, not significant.

To validate the cell‐autonomous role of NMI, we differentiated brown adipocytes in vitro from the BAT SVF of *Nmi^−/−^* mice (Figure S4B,C). *Nmi* deficiency led to reduced lipid accumulation (Figure 6A), suggestive of a more oxidative metabolic state. Based on transcriptomic data pointing to PPAR signaling, we next investigated whether this pathway was necessary for the enhanced adaptive thermogenesis in *Nmi^−/−^* adipocytes. Pharmacological inhibition of PPARα with GW6471 or PPARγ with GW9662 suppressed PPAR pathway activation and UCP1 induction (Figure 6B–E, Figure S4D,E). Furthermore, siRNA‐mediated knockdown of *Ppara* specifically reversed the elevated UCP1 expression (Figure 6F, Figure S4F,G). Collectively, these loss‐of‐function experiments demonstrate that PPAR signaling, particularly PPARα, is required for the augmented thermogenic capacity resulting from *Nmi* deletion. These findings provide a mechanistic basis for the increased energy expenditure observed in *Nmi^−/−^* mice (Figure 6G).

### 2.7 NMI‐Mediated TLR4/IRF3 Signaling Impairs Adipocyte Thermogenesis

**FIGURE 7:**
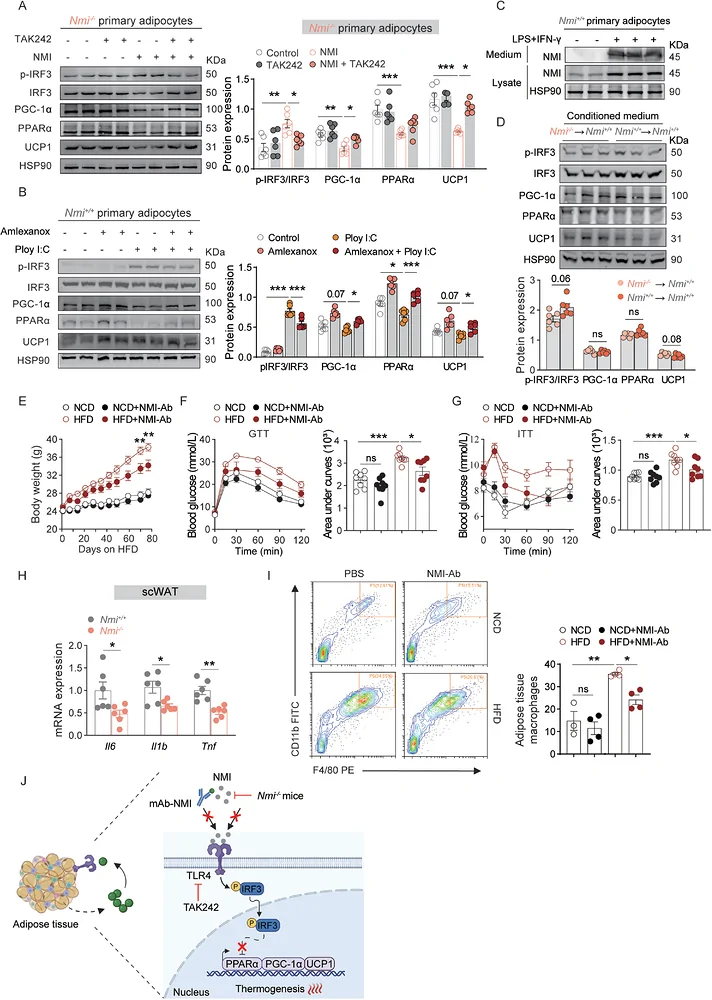
NMI suppresses adaptive thermogenesis through TLR4/IRF3 signaling. (A) Mature adipocytes were pretreated with TAK‐242 (1 µm, TLR4 inhibitor) for 2 h, followed by recombinant NMI (5 µg mL^−1^) treatment for 24 h. Thermogenic proteins were subsequently analyzed by Western blotting (*n* = 6). (B) Mature adipocytes were pretreated with Amlexanox (50 µm, IRF3 inhibitor) for 1 h, followed by Poly I:C (50 µg mL^−1^, 1 h) treatment to induce IRF3 phosphorylation. Cell lysates were collected for detection of the indicated proteins (*n* = 6). (C) Mature adipocytes were stimulated with IFN‐γ (20 ng mL^−1^) and LPS (100 ng mL^−1^) for 24 h. Culture supernatants were analyzed for NMI secretion (*n* = 3). (D) Conditioned media from stimulated *Nmi^+/+^* and *Nmi^−/−^* adipocytes were transferred to WT primary adipocytes. Expression of the indicated proteins was subsequently examined by Western blotting (*n* = 6). (E) BW gain in DIO mice treated with monoclonal antibody‐mediated neutralization of NMI (mAb‐NMI, 10 mg kg^−1^) or PBS (*n* = 8). (F, G) GTT (F) and ITT (G) in DIO mice after 12 weeks of HFD and mAb‐NMI or PBS treatment, quantified by AUC (*n* = 8). (H) HFD‐induced adipose tissue inflammation in *Nmi^+/+^* and *Nmi^−/−^* mice (*n* = 6). (I) Macrophage infiltration in DIO mice treated with mAb‐NMI or PBS (*n* = 3–4). (J) A model summarizing that NMI, secreted by adipose tissue under HFD‐induced metabolic stress, activates TLR4/IRF3 signaling in brown adipocytes to suppress adaptive thermogenesis (Created with BioRender.com). Data are represented as mean ± SEM. Statistical analyses: one‐way ANOVA followed by Bonferroni's multiple comparisons test (A, B, I, AUC of F, G), two‐tailed unpaired Student's t‐test (D, H), two‐way ANOVA followed by Bonferroni's multiple comparisons test (E–G). *p < 0.05, **p < 0.01, ***p < 0.001; ns, not significant.

As NMI functions as a TLR4 agonist and TLR4/IRF3 signaling suppresses adipocyte adaptive thermogenesis [22, 23, 38, 39, 40, 41], we hypothesized that NMI impairs PPAR‐mediated adaptive thermogenesis through this axis. Stimulation of mature adipocytes with recombinant NMI induced IRF3 phosphorylation and downregulated key thermogenic proteins (PPARα, PGC‐1α, UCP1). These effects were alleviated by co‐treatment with the TLR4 inhibitor TAK‐242 (Figure 7A, Figure S5A). Next, we investigated the link between IRF3 and repression of the PPARα/PGC‐1α axis by pharmacologically modulating IRF3 activity. IRF3 activation induced by a synthetic double‐stranded RNA analog (Poly I:C) suppressed thermogenic gene expression and reduced PPARα, PGC‐1α, and UCP1 levels, whereas pharmacological inhibition of IRF3 signaling with the small‐molecule inhibitor (Amlexanox) alleviated these effects (Figure 7B). These results demonstrate that TLR4/IRF3 signaling is a critical mediator of NMI‐induced thermogenic suppression.

Given that activated macrophages release NMI [30, 31] and adipocytes also secrete other DAMPs [42, 43, 44], we investigated whether adipocytes themselves produce NMI. In an in vitro model mimicking an HFD‐induced inflammatory environment, adipocytes expressed and secreted NMI in response to interferon‐gamma (IFN‐γ) and lipopolysaccharide (LPS) stimulation (Figure 7C). To test for autocrine function, we performed conditioned medium transfer experiments. Wild‐type adipocyte‐derived medium induced stronger IRF3 activation and PPARα/PGC‐1α/UCP1 suppression than medium from *Nmi^−/−^* adipocytes, although this difference did not reach statistical significance (Figure 7D). Together with the significant effects of exogenous NMI, these data suggest that NMI‐mediated suppression of adipocyte adaptive thermogenesis may involve not only adipocyte‐derived signals but also contributions from other cell types in adipose tissue, such as macrophages [30].

To evaluate the therapeutic potential of targeting NMI, we administered an NMI‐neutralizing antibody to DIO mice (Figure S5B–D). This treatment significantly reduced BW gain, improved glucose tolerance and insulin sensitivity, as well as attenuated lipid accumulation (Figure 7E–G, Figure S5E). Furthermore, *Nmi* deficiency significantly attenuated HFD‐induced WAT inflammation, as shown by reduced levels of key inflammatory cytokines (*Il6*, *Il1b*, *Tnf*) in *Nmi^−/−^* mice (Figure 7H). Similarly, NMI neutralization significantly reduced macrophage infiltration (F4/80^+^ CD11b^+^) in WAT (Figure 7I, Figure S5F), indicating ameliorated adipose tissue inflammation.

In summary, our findings delineate a pathway in which adipose tissue inflammatory stress induces NMI secretion, which in turn activates adipocyte TLR4/IRF3 signaling to suppress PPAR‐mediated adaptive thermogenesis, thereby promoting obesity (Figure 7J). This mechanism highlights the role of NMI as a key link between inflammatory signaling and energy metabolism.

## 3 Discussion

Obesity is characterized by chronic low‐grade inflammation that disrupts adipose tissue homeostasis and contributes to metabolic dysfunction [11]. Although thermogenic activation has been shown to counteract inflammation and improve systemic metabolism [21, 45, 46], the endogenous signals that suppress this process in obesity remain poorly understood. In this study, we identify NMI as an adipose tissue‐derived regulator that links inflammatory signaling to impaired thermogenic capacity. Our findings demonstrate that under dietary and inflammatory stress, adipose tissue‐secreted NMI engages TLR4, leading to IRF3 phosphorylation and subsequent inhibition of adaptive thermogenesis. Thus, NMI emerges as a previously unrecognized negative regulator of adaptive thermogenesis and a critical node connecting adipose inflammation to impaired energy expenditure.

NMI belongs to the DAMP family of endogenous molecules, many of which are upregulated in obesity and perpetuate sterile inflammation through TLR4 signaling [15, 47]. TLR4 activation in adipose tissue disrupts metabolic function by promoting insulin resistance and suppressing UCP1 expression [23, 40], whereas its inhibition ameliorates inflammation [48]. Unlike classical DAMPs such as HMGB1 [19, 44, 49, 50, 51] and HSP70/90 [52, 53, 54], which modulate energy metabolism via the cAMP/PKA pathway [49, 52, 53, 55, 56] or PPARγ activity [57], NMI exhibits distinct characteristics [19, 20]. NMI is selectively upregulated under chronic metabolic stress and is secreted from living adipocytes in a regulated manner, rather than being passively released during cell death. In contrast to its induction under metabolic stress, *Nmi* expression is preferentially and persistently downregulated upon cold exposure. Moreover, our data indicate that NMI signals through TLR4 with a downstream bias toward IRF3 activation, suggesting that NMI selectively suppresses adaptive thermogenesis compared with other DAMPs that more broadly activate NF‐κB‐dependent inflammatory pathways.

Elevated circulating levels of NMI in obese miniature pigs [58] and nonhuman primates [59] further support its physiological relevance in energy homeostasis. Importantly, multiple lines of evidence support a non‐redundant and potentially prominent role for NMI in vivo. Genetic and pharmacological modulation of NMI leads to measurable changes in adipose thermogenic capacity and systemic energy expenditure, supporting a functional role for NMI that may not be fully compensated for by other DAMPs. Collectively, these findings position NMI as a context‐dependent, functionally distinct adipose tissue‐derived DAMP that links sterile inflammation to impaired energy expenditure. While partial redundancy with other DAMPs cannot be excluded, our data support a unique and potentially prominent role for NMI in suppressing adaptive thermogenesis under metabolic stress.

Mechanistically, we delineate a pathway in which adipose tissue‐derived NMI activates the TLR4/IRF3 axis, thereby suppressing PPARα‐mediated thermogenic transcription. TLR4 activation in adipocytes initiates a TRIF‐dependent cascade that leads to IRF3 activation [21, 38, 41], an axis known to promote inflammation and insulin resistance while directly inhibiting browning and thermogenesis [22, 23, 38, 40]. Our findings identify that NMI serves as an upstream activator of this axis. Furthermore, NMI suppresses the PPARα/PGC‐1α/UCP1 transcriptional program, a core regulatory hub for adaptive thermogenesis and mitochondrial biogenesis [60, 61]. Consistent with this, *Nmi* deficiency enhances PPAR signaling and upregulates thermogenic genes, whereas exogenous NMI reverses these effects in a TLR4‐mediated manner.

Beyond this linear pathway, our data suggest a self‐amplifying circuit that perpetuates thermogenic suppression under metabolic stress. Inflammatory stimulation induces the intracellular accumulation and secretion of NMI from adipocytes, and secreted NMI subsequently activates TLR4. In turn, TLR4 activation and the ensuing inflammatory response may further enhance NMI expression and secretion, creating an autocrine or paracrine loop that sustains IRF3 phosphorylation and transcriptional repression of the PPARα/PGC‐1α/UCP1 axis. This positive feedback mechanism provides a mechanistic explanation for the persistent impairment of adaptive thermogenesis in obese adipose tissue.

The therapeutic relevance of targeting NMI is supported by genetic and pharmacological intervention studies. Both genetic ablation of *Nmi* and pharmacological neutralization with a monoclonal antibody protected mice from DIO, improved insulin sensitivity, and attenuated adipose tissue inflammation. Notably, NMI neutralization reduced macrophage infiltration, a hallmark of adipose tissue inflammation, suggesting that targeting NMI may simultaneously alleviate inflammation and ameliorate metabolic dysfunction. Together, these findings provide proof‐of‐concept evidence that targeting NMI can modify the metabolic phenotype and support a causal role for NMI in its development.

Several limitations of this study should be acknowledged. First, although our data indicate that enhanced energy expenditure is the primary driver of the lean phenotype in *Nmi*‐deficient mice, minor contributions from other factors, such as alterations in nutrient absorption, fecal energy loss, or the gut microbiome, cannot be completely excluded. Second, while we have delineated NMI's role in repressing adaptive thermogenesis through the TLR4/IRF3 pathway and suppression of the PPARα/PGC‐1α/UCP1 transcriptional program, the precise mechanism by which NMI regulates PPARα transcriptional activity remains incompletely defined. Based on existing evidence, we propose two plausible regulatory pathways: modulation of STAT3 signaling [62], which may enhance fatty acid oxidation via PPARα [63, 64] and promote PPARγ expression via C/EBPβ [65, 66], and interaction with IRE1α, where *Nmi* deficiency may alleviate IRE1α‐mediated suppression of PGC‐1α, thereby augmenting PPARα activity [25, 26, 27, 28, 29]. Third, although the increased energy expenditure is primarily attributed to BAT activation and scWAT browning, we acknowledge that other tissues, such as skeletal muscle, may also contribute. Notably, *Nmi* expression in skeletal muscle did not change significantly after HFD feeding; nevertheless, tissue ‑ specific studies will be required to fully delineate the contribution of individual tissues. Similarly, while whole‐body RER showed no significant difference, tissue‐specific changes in substrate utilization cannot be excluded. Additionally, while TLR4 serves as a key receptor for extracellular NMI, the potential involvement of other receptors such as TLR2 or receptor for advanced glycation end‐products (RAGE) cannot be ruled out, and the cellular mechanisms governing NMI secretion remain undefined. Finally, the use of global *Nmi* knockout mice limits tissue‑specific interpretations. Future studies employing cell‐type‐specific genetic models will be essential to dissect the physiological role of NMI signaling in whole‐body energy homeostasis, to more precisely define the cellular source and mode of action of NMI, and to evaluate its potential as a therapeutic target for obesity.

In conclusion, our work positions NMI as a pivotal immunometabolic regulator linking sterile inflammation to impaired energy expenditure. We delineate a pathway in which adipose tissue‐derived NMI activates the TLR4/IRF3 axis, thereby suppressing the PPARα/PGC‐1α/UCP1 transcriptional program and inhibiting adaptive thermogenesis. This regulatory mechanism not only advances our understanding of obesity pathogenesis but also identifies NMI as a promising therapeutic target. Pharmacological disruption of this signaling pathway may restore thermogenesis and offer a novel strategy to combat obesity and its associated metabolic disorders.

## 4 Experimental Methods

### 4.1 Gene Expression Analysis in Clinical Cohorts and Mouse Bioinformatic Analysis

The initial transcriptomic profiles were derived from the NCBI Gene Expression Omnibus (GEO, https://www.ncbi.nlm.nih.gov/geo/) and included subcutaneous white adipose tissue (scWAT) biopsies of 770 male participants in the population‐based METSIM study. These profiles were obtained using the Affymetrix Human Genome U219 Array and deposited under accession number GSE70353. Additional transcriptomic data came from abdominal scWAT biopsies of 25 males and 28 females with overweight or obesity (BMI: 28–35 kg m^−2^) who underwent a dietary intervention; these transcriptome profiles were acquired using the Affymetrix Human Gene 1.1 ST Array, and the dataset was archived under accession number GSE77962.

Bioinformatic analysis was performed using mouse datasets sourced from the NCBI Gene Expression Omnibus (GEO, https://www.ncbi.nlm.nih.gov/geo/). The GSE15822 dataset, which was generated using the Illumina Mouse‐6 v1.1 expression bead chip and contains data from various adipose tissues of mice fed a standard breeding diet, was analyzed. In addition, the GSE242711 dataset (Illumina NextSeq 500 platform) and the GSE294416 dataset (DNBSEQ‐G50 platform) were analyzed by RNA sequencing to assess mRNA levels in mature adipocytes from the brown, beige, and white adipose depots of mice exposed to cold for 21 days and 24 h, respectively.

### 4.2 Animal Experiments

All animal experiments were conducted in accordance with the Guide for the Care and Use of Laboratory Animals and were approved by the Institutional Animal Care and Use Committee (IACUC) of Sun Yat‐sen University (Approval No. SYSU‐IACUC‐2023‐001743). *Nmi* knockout (referred to as *Nmi^−/−^*) mice on a C57BL/6J background were generated via CRISPR‐Cas9‐mediated gene editing as previously described [30]. The mice were housed in a specific pathogen‐free (SPF) facility at the Experimental Animal Center of Sun Yat‐sen University under controlled environmental conditions (temperature: 23°C ± 2°C; humidity: 50% ± 10%; 12‐h light/dark cycle), with free access to standard chow diet and water. Genotyping was performed by PCR using the primer sequences listed in Table S1.

### 4.3 Diet‐Induced Obesity (DIO) Mouse Models

Male *Nmi* knockout (*Nmi^−/−^*) mice and their wild‐type littermate controls (referred to as wild‐type, WT or *Nmi^+/+^*) were generated as described above. Littermate mice of the same sex and age were used as controls in all experiments. Unless otherwise specified, mice were maintained at ambient room temperature (23°C ± 2°C) under a 12‐h light/dark cycle, with free access to water and food. *Nmi^−/−^* and *Nmi^+/+^* mice were fed a standard chow diet until 8 weeks of age and then randomly assigned to either a normal chow diet (NCD) group or a high‐fat diet (HFD) group. The HFD contained 60% kcal from fat, 20% kcal from carbohydrate, and 20% kcal from protein (5.42 kcal g^−1^; D12492; Research Diets, USA). The control NCD contained 10% kcal from fat (3.85 kcal g^−1^; D12450J; Research Diets, USA). Both diets were fed for 12 consecutive weeks. Body weight (BW) was measured weekly at the same time of day.

### 4.4 Intraperitoneal Glucose Tolerance Test (GTT) and Insulin Tolerance Test (ITT)

The GTT and ITT were performed at designated time points. Mice were fasted for 15 h (from 18:00 to 09:00) prior to IPGTT or for 6 h (from 09:00 to 15:00) prior to ITT. For GTT, glucose (2.0 g kg^−1^ BW; Sigma–Aldrich, USA) dissolved in sterile saline was administered by intraperitoneal injection. For ITT, insulin (0.75 U kg^−1^ BW; Novolin R, Denmark) was administered by intraperitoneal injection. Blood glucose levels were measured from the tail vein at baseline (0 min) and at 15‐, 30‐, 60‐, 90‐, and 120‐min post‐injection using a digital glucometer (Yuwell, China). The area under the curve (AUC) was calculated [67].

### 4.5 Energy Metabolism

Mice were individually housed in metabolic cages and acclimated for 24 h prior to data collection. To minimize stress‐induced artifacts, mice were first habituated to the experimental room for 3 days and then to the metabolic cages for 24 h, with the acclimation period excluded from analysis. Energy metabolism parameters were measured using a Promethion metabolic system (Sable Systems International, USA). The 48‐h measurement period was designed to ensure a stable metabolic state [68]. During the measurement period, oxygen consumption (VO_2_), carbon dioxide production (VCO_2_), respiratory exchange ratio (RER), food intake, and locomotor activity were recorded continuously at 5‐min intervals. As previously described, VO_2_, VCO_2_, and energy expenditure were normalized to BW [69, 70].

### 4.6 Food Intake

Spontaneous food intake was measured in free‐feeding, individually caged mice after a 24‐h acclimation period, as previously described [69]. At the beginning of the measurement period, an equal amount of fresh food (corresponding to each mouse's respective diet: NCD or HFD) was provided to each mouse. After 24 h, food remnants and spillage were collected and weighed. Food intake was calculated by subtracting the weight of the remnants and spillage from the initial food amount provided.

### 4.7 Body Composition

Body composition, including fat mass and lean mass, was evaluated at the end of the 12‐week dietary intervention using magnetic resonance imaging (EchoMRITM‐100, USA) according to the manufacturer's instructions. Mice were measured in the conscious state without anesthesia. The instrument was calibrated daily using a standard reference sample provided by the manufacturer.

### 4.8 Thermoneutrality and Cold Exposure Tests

Male *Nmi^−/−^* and *Nmi^+/+^* mice aged 8 weeks were individually housed at thermoneutrality (30°C) for 3 days prior to acute cold exposure. For cold exposure, mice were transferred to a 4°C environmental chamber without food but with free access to drinking water. Core body temperature was measured serially at 0 (baseline), 1, 2, 6, 12, and 24 h after cold exposure initiation using a rectal thermometer (FT3400, China). The probe was inserted 2 cm into the rectum with a small amount of lubricant. Surface temperature was also assessed at the 24‐h endpoint using a Fluke Ti480U infrared thermography camera (Fluke Corporation, USA) in a non‐invasive manner. Mice were euthanized if body temperature decreased by 10°C from baseline in accordance with IACUC‐approved humane endpoints.

### 4.9 RNA Extraction and Real‐Time Quantitative PCR (qPCR)

At the end of the experiments, mice were euthanized. Following confirmation of death, brown adipose tissue (BAT), scWAT, epididymal white adipose tissue (eWAT), perirenal white adipose tissue (pWAT), and the liver were rapidly excised, weighed, frozen in liquid nitrogen, and stored at −80°C for subsequent analyses.

Total RNA was extracted from tissues or cells using TRIzol reagent (Invitrogen, USA). RNA concentration and purity were assessed using a NanoDrop spectrophotometer (Thermo Fisher Scientific, USA), and RNA integrity was verified by agarose gel electrophoresis. Subsequently, 1 µg of RNA was reverse‐transcribed into cDNA using the PrimeScript RT kit according to the manufacturer's protocol. qPCR was performed using SYBR Green Master Mix on a QuantStudio 7 Flex Real‐Time PCR System (Thermo Fisher Scientific, USA). Gene expression levels were normalized to the housekeeping gene Rn18s (18S ribosomal RNA) using the ΔΔCt method and are expressed as fold changes relative to the control. Primer sequences are listed in Table S2.

### 4.10 RNA Sequencing and Data Analysis

RNA sequencing was outsourced to Berry Genomics Co., Ltd. The RNA quality of BAT from *Nmi^+/+^* and *Nmi^−/−^* mice (*n* = 4 per genotype) was assessed using an Agilent TapeStation prior to library preparation. Sequencing libraries were constructed using the TruSeq RNA Sample Prep Kit v2 (Illumina) according to the manufacturer's instructions. Library quality and fragment size were verified using an Agilent 2100 Bioanalyzer (Agilent). The normalized libraries were then pooled and sequenced on an Illumina NovaSeq 6000 platform to generate 150 bp single‐end reads with barcode multiplexing, generating approximately 60 million reads per sample. RNA‐seq data analysis was performed using the OmicShare platform (https://www.omicshare.com/tools) [71].

### 4.11 Histological and Morphological Analysis

Tissue samples of liver and adipose tissue were fixed in 4% paraformaldehyde (PFA) for 24 h, embedded in paraffin, and sectioned into 4‐µm slices. After deparaffinization in xylene and rehydration through a graded ethanol series, sections were stained with hematoxylin and eosin (H&E) for general morphological observation. For immunohistochemical analysis, the sections were subjected to antigen retrieval in citrate buffer (pH 6.0) at 95°C–100°C for 20 min. Subsequently, endogenous peroxidase activity was quenched by incubating the sections with 3% H_2_O_2_ for 15 min, followed by blocking with 5% goat serum for 1 h. The sections were then incubated with a primary antibody overnight at 4°C. Staining was performed using an ABC kit (Vector Laboratories, USA), followed by counterstaining with Harris hematoxylin. Images were acquired using a ZEISS microscope (Axio Imager Z2, Germany).

### 4.12 Western Blot Analysis and Antibodies

Tissues or cells were lysed in RIPA lysis buffer (Beyotime Biotechnology,China) containing a protease inhibitor cocktail (Roche, Indianapolis, IN). Total protein concentration was determined using a bicinchoninic acid (BCA) assay kit (Beyotime Biotechnology, China). Equal amounts of protein were boiled in loading buffer and separated on 10% or 12.5% SDS‐polyacrylamide gels, followed by transfer onto 0.2 µm PVDF membranes (Immobilon‐PSQ, Merck). The membranes were blocked with 5% non‐fat milk or bovine serum albumin (BSA) for 1 h and then incubated with primary antibodies overnight at 4°C. After washing three times with TBST (10 min per wash), the membranes were incubated with HRP‐conjugated secondary antibodies for 1–2 h at room temperature. The antibodies used were: α‐tubulin (Ray antibody, Cat#RM2007), β‐actin (Proteintech, Cat#66009), UCP1 (Abcam, Cat#ab209483), HSP90 (Proteintech, Cat#13171‐1‐AP), PPARα (Proteintech, Cat#66826), PPARγ (Cell Signaling Technology, Cat#2435), NMI (Servicebio, Cat#GB114586), p‐IRF3 (Cell Signaling Technology, Cat#29047), IRF3 (Cell Signaling Technology, Cat#4302), Anti‐rabbit IgG HRP‐linked Antibody (Cell Signaling Technology, 7074S), Anti‐mouse IgG HRP‐linked Antibody (Cell Signaling Technology, 7076S). After incubation with the HRP‐conjugated secondary antibodies, reactive bands were visualized using an ECL Plus Western Blotting Kit (Pierce Biotechnology, USA) and an ultrasensitive multifunctional iBright 1500 Imaging System (Thermo Fisher Scientific, USA). The relative intensity of the reactive bands was analyzed using ImageJ software.

### 4.13 Primary Adipocyte Culture

Adipose tissue was isolated from 4 to 5‐week‐old male *Nmi^−/−^* mice and their *Nmi^+/+^* littermate controls and was immediately minced according to a previously described methods for isolating and culturing primary stromal vascular fraction (SVF) cells [72, 73, 74]. The tissue fragments were then digested with type II collagenase (Sigma, USA) for 30–45 min to obtain a homogeneous single‐cell suspension, which was subsequently filtered through a 40‐µm cell strainer. After centrifugation at 500–600 × g for 10 min at 4°C, the cell pellet was suspended in complete Dulbecco's Modified Eagle Medium (DMEM, Gibco, USA) supplemented with 10% fetal bovine serum (FBS) and 1% penicillin‐streptomycin and seeded onto culture plates. The differentiation program for the SVF cells was initiated according to the previously described method [73].

### 4.14 Cell Culture

The mouse‐derived 3T3‐L1 preadipocyte cell line was purchased from the American Type Culture Collection (ATCC, CL‐173, USA). Cells were maintained in DMEM (Gibco, USA) supplemented with 10% FBS and 1% penicillin‐streptomycin. Cells were grown at 37°C in a humidified incubator with 5% CO_2_.

### 4.15 Adipocyte Differentiation

3T3‐L1 preadipocytes or primary adipocytes (passages ≤ P4) were induced to differentiate 2 days after reaching confluence, with this time point designated as Day 0. The differentiation induction medium contained insulin (5 µg mL^−1^), dexamethasone (1 µm), 3‐isobutyl‐1‐methylxanthine (0.5 mm), indomethacin (125 nm), triiodothyronine (1 nm), and rosiglitazone (1 µm). From Day 2, the medium was replaced with maintenance medium containing insulin (5 µg mL^−1^), triiodothyronine (1 nM), and rosiglitazone (1 µm), and was changed every other day. Fully differentiated adipocytes were harvested on Days 6–8.

### 4.16 In Vitro Compounds Treatment

To investigate the roles of PPARα, PPARγ, IRF3 signaling, differentiated mature adipocytes were treated with GW6471 (a PPARα antagonist; Selleck, Cat#S2798, 10 µm), GW9662 (a PPARγ antagonist; Selleck, Cat#S2915, 10 µm), poly(I:C) (an IRF3 phosphorylation inducer; MedChemExpress, Cat#HY‐107202, 50 mg mL^−1^), or amlexanox (an IRF3 inhibitor; MedChemExpress, Cat#HY‐B0713, 50 µm) for 24–48 h (GW6471 and GW9662) or 1 h (poly(I:C) and amlexanox), after which the cells were harvested for RNA and protein analysis.

### 4.17 Recombinant NMI Protein

The full‐length mNMI protein was expressed in *E. coli* strain BL21 (DE3). Bacterial cells were harvested by centrifugation at 1500 × g for 10 min post‐expression. The pellet was resuspended in lysis buffer (20 mm Tris at pH 8.0, 400 mM NaCl) and lysed by sonication. The lysate was then centrifuged at 12 000 × g for 30 min to separate insoluble debris. The clarified supernatant was applied to a Ni‐NTA affinity column (Qiagen) and washed extensively with elution buffer (20 mm Tris at pH 8.0, 150 mm NaCl, 50 mm imidazole). The recombinant protein was eluted using an elution buffer (20 mm Tris at pH 8.0, 150 mm NaCl, 500 mm imidazole). Further purification was performed by gel filtration on a Superdex 200 column (Cytiva) using the lysis buffer on an FPLC system, as described previously [30]. Endotoxins were subsequently removed via PMB chromatography.

### 4.18 Oil Red O Staining

Oil Red O staining was performed as previously reported [9]. A 0.5% (mg v^−1^) Oil Red O stock solution in isopropanol was diluted with water (3:2) and filtered through a 0.45‐µm filter. Differentiated adipocytes were washed three times with PBS and fixed with 4% PFA for 30 min. The fixed cells were then incubated with the filtered Oil Red O solution at room temperature for 1 h to visualize lipid droplets. After washing with PBS, 100% isopropanol was added as an extraction solvent to elute the stained dye from the cells. The absorbance of the extracted dye was measured at 510 nm using a BioTek Epoch2 microplate reader.

### 4.19 Nmi Neutralizing Antibody Treatment

Monoclonal antibody‐mediated neutralization of NMI (mAb‐NMI) was prepared in the laboratory according to the classical monoclonal hybridoma technique [31, 75]. Each mouse was intravenously injected with 100 µg (10 mg kg^−1^) mAb‐NMI in 100 µL sterile phosphate‐buffered saline (PBS). The injections were administered twice weekly, commencing at the onset of the dietary intervention.

### 4.20 Flow Cytometry Sorting

Single‐cell suspensions were prepared from mice fed either an NCD or HFD according to the aforementioned SVF isolation method [72, 73, 74] and filtered through a 40‐µm cell strainer. Cells were resuspended in PBS containing 1% BSA and stained with the specified antibodies for 30 min on ice. For intracellular marker staining, cells were first fixed with fixation buffer (Biolegend, USA, Cat#420801) at 4°C for 30 min, followed by incubation with antibodies diluted in intracellular permeabilization wash buffer (Biolegend, USA, Cat#421002). The following antibodies were used: FITC‐conjugated anti‐mouse CD45 (Cat#157214) and PerCP/Cy5.5‐conjugated anti‐mouse F4/80 (Cat#123128). Cell sorting was performed using a CytoFLEX SRT cell sorter (Beckman, USA). The corresponding CD45‐positive (CD45^+^) and CD45‐negative (CD45^−^) cell populations were collected and analyzed using FlowJo software (v10.5.3).

### 4.21 Statistical Analysis

All data are presented as the mean ± standard error of the mean (SEM). Unless otherwise stated, all in vitro studies were performed with at least three independent replicates. For qPCR data, normalization was performed using the ΔΔCt method with *Rn18s* as the internal reference gene. Sample sizes (n) for each statistical analysis are indicated in the respective figure legends. Depending on the experimental design, statistical significance was assessed using paired or unpaired two‐tailed Student's t‐tests for two‐group comparisons, or one‐way or two‐way analysis of variance (ANOVA) followed by Bonferroni's post hoc test where appropriate for multiple comparisons. The significance level (alpha value) was set at 0.05. A *p*‐value of less than 0.05 was considered statistically significant (*p < 0.05, **p < 0.01, and ***p < 0.001; ns, not significant). All statistical analyses were performed using GraphPad Prism 9 software.

## Author Contributions

**Ting‐Ting Li**: conceptualization, formal analysis, software, data curation, methodology, writing – original draft, and writing – review and editing. **Xin‐Yuan Zhao**: conceptualization, formal analysis, software, data curation, methodology, writing – review and editing. **Min Zhang**: conceptualization, formal analysis, software. **Zhuang‐Feng Weng**: conceptualization, formal analysis, software, funding acquisition. **Xiao‐Ran Guo**: software, data curation. **Ying‐Fang Liu**: conceptualization, formal analysis, software, data curation, methodology, writing – review and editing, funding acquisition, project administration. **Qiao‐Ping Wang**: conceptualization, formal analysis, software, data curation, methodology, writing – review and editing, funding acquisition, project administration. **Huan‐Huan Liang**: conceptualization, formal analysis, software, data curation, methodology, writing – review and editing, funding acquisition, project administration.

## Fundings

This work was supported by National Key Program of China (project No. 2022YFE0210000, 2023YFC2606400), Shenzhen Science and Technology Planning Projec (ZDSYS20220606100803007), National Natural Science Foundation of China (82373883, 82071346, 32271321), Pearl River Talent Plan Innovation and Entrepreneurship Team Project of Guangdong Province (2019ZT08Y464), Technology and Innovation Bureau of Shenzhen Municipality (202109293000005), Key Fundamental Research Projects of Shenzhen Science and Technology Plan (JCYJ20220818102017035), Natural Science Foundation of Guangdong Province (2023A1515010245), Shenzhen Medical Research Fund (D2403006), Science and Technology Innovation Committee of Shenzhen, China (JCYJ20240813151248062).

## Ethics Approvals

All animal experiments were conducted in accordance with the Guide for the Care and Use of Laboratory Animals and were approved by the Institutional Animal Care and Use Committee (IACUC) of Sun Yat‐sen University (Approval No. SYSU‐IACUC‐2023‐001743).

## Conflicts of Interest

The authors declare no conflicts of interest.

## Supporting information




**Supporting File**: advs75921‐sup‐0001‐SuppMat.docx.

## Data Availability

The data that support the findings of this study are available from the corresponding author upon reasonable request.
