# Supplementary material for: NMI Regulates Adipose Adaptive Thermogenesis Through TLR4/IRF3 Signaling to Promote Obesity
Source: Adv Sci (Weinh). 2026 Jun 2:e75921. Online ahead of print. doi: 10.1002/advs.75921 (PMC13337080; doi:10.1002/advs.75921)
Supplement: Supplementary file 1 — Supporting file: advs75921‐sup‐0001‐SuppMat.docx [file ADVS-9999-e75921-s001.docx]

**Supplemental information**

**NMI Regulates Adipose Adaptive Thermogenesis Through TLR4/IRF3 Signaling to Promote Obesity**

Ting-Ting Li^1,2^, Xin-Yuan Zhao^3,4,5^, Min Zhang^1,2^, Zhuang-Feng Weng^1,2^, Xiao-Ran Guo^1,2^, Ying-Fang Liu^1,2^*, Qiao-Ping Wang^3,4,6^*, Huan-Huan Liang^1,3^*


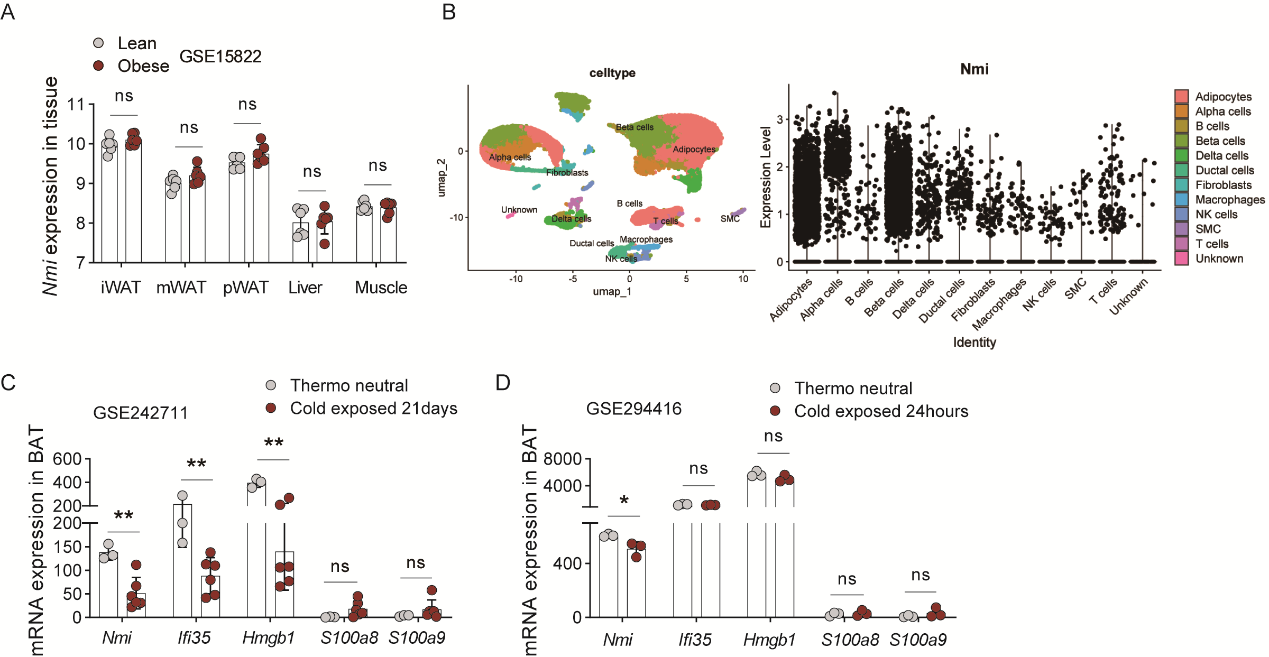


**Figure S1. NMI expression is regulated by metabolic stress and cold exposure in mice.**

(A) *Nmi* mRNA expression in various tissues from lean and obese mice (n = 6).

(B) Uniform manifold approximation and projection (UMAP) plot showing major cell populations in adipose tissue from single-nucleus RNA sequencing (snRNA-seq) data (GSE296575, n = 8,000 nuclei).

(C, D) DAMP mRNA expression in NCD-fed mice after 21 days (C, n = 6) or 24 hours (D, n = 3) of cold exposure (4 °C).

Data are represented as mean ± SEM. Statistical analyses: two-tailed unpaired Student’s t-test (A, C, D). **p* < 0.05, ***p* < 0.01, ****p* < 0.001; ns, not significant.


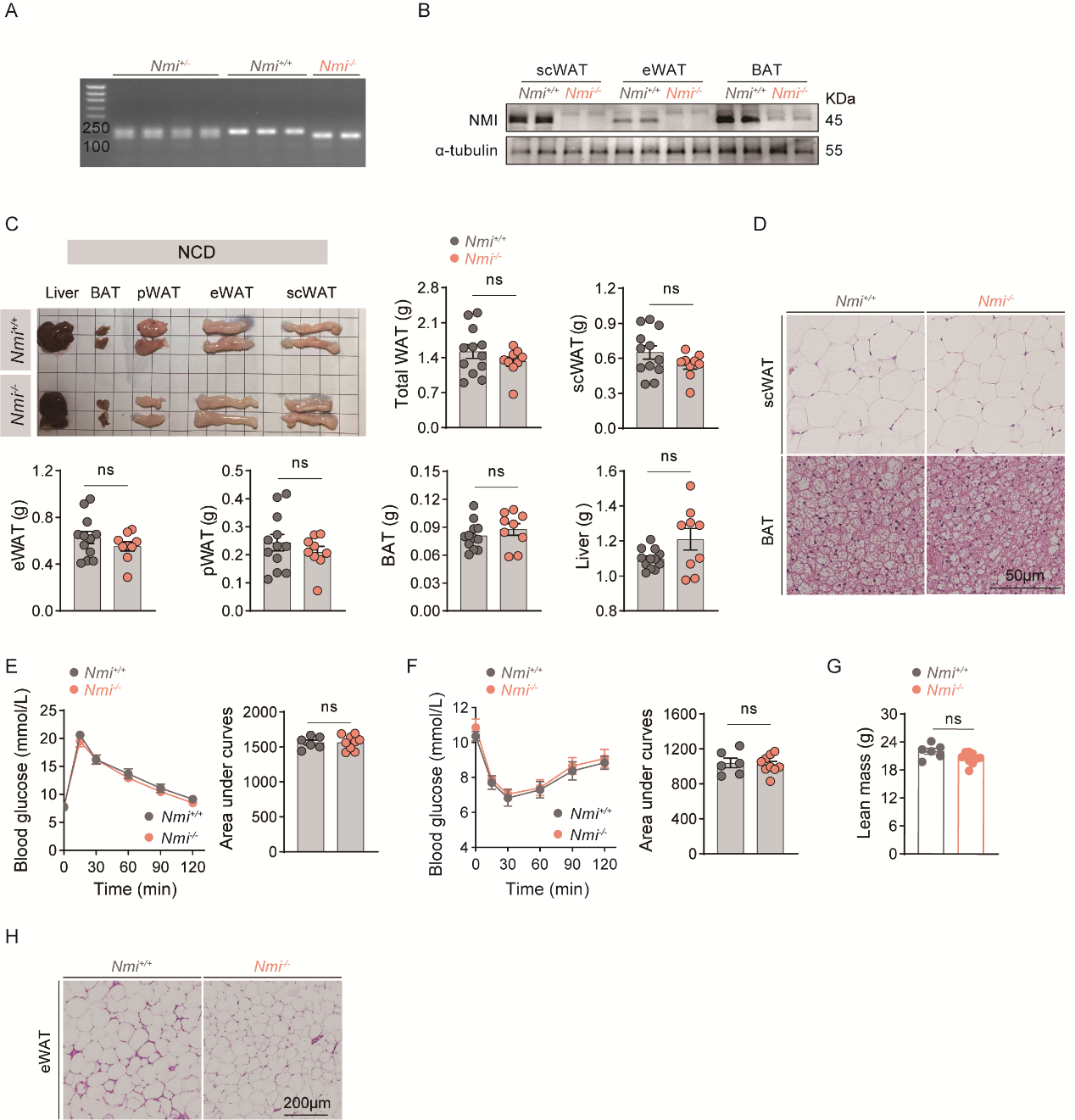


**Figure S2. *Nmi* deficiency does not affect energy balance under NCD feeding.**

(A, B) Confirmation of *Nmi* knockout at DNA (A) and protein (B) levels in different adipose tissues.

(C) Representative images and weights of various adipose tissues and liver from *Nmi^+/+^* and *Nmi^-/-^* mice after 12 weeks of NCD feeding (n = 9-12).

(D) Representative images of H&E staining in subcutaneous white adipose tissue (scWAT) and brown adipose tissue (BAT) after 12 weeks of NCD feeding.

(E, F) Glucose tolerance test (GTT, E) and insulin sensitivity test (ITT, F) in *Nmi^+/+^* and *Nmi^-/-^* mice under NCD conditions, quantified by area under the curve (AUC) (n = 6-9).

(G) Lean mass of *Nmi^+/+^* and *Nmi^-/-^* mice after 12 weeks of HFD feeding, measured by nuclear magnetic resonance (NMR) (n = 6-9).

(H) Representative images of H&E staining in eWAT from *Nmi^+/+^* and *Nmi^-/-^* mice after 12 weeks of HFD feeding.

Data are represented as mean ± SEM. Statistical analyses: two-tailed unpaired Student’s t-test (C, E-G), two-way ANOVA followed by Bonferroni’s multiple comparisons test (E, F). **p* < 0.05, ***p* < 0.01, and ****p* < 0.001; ns, not significant.


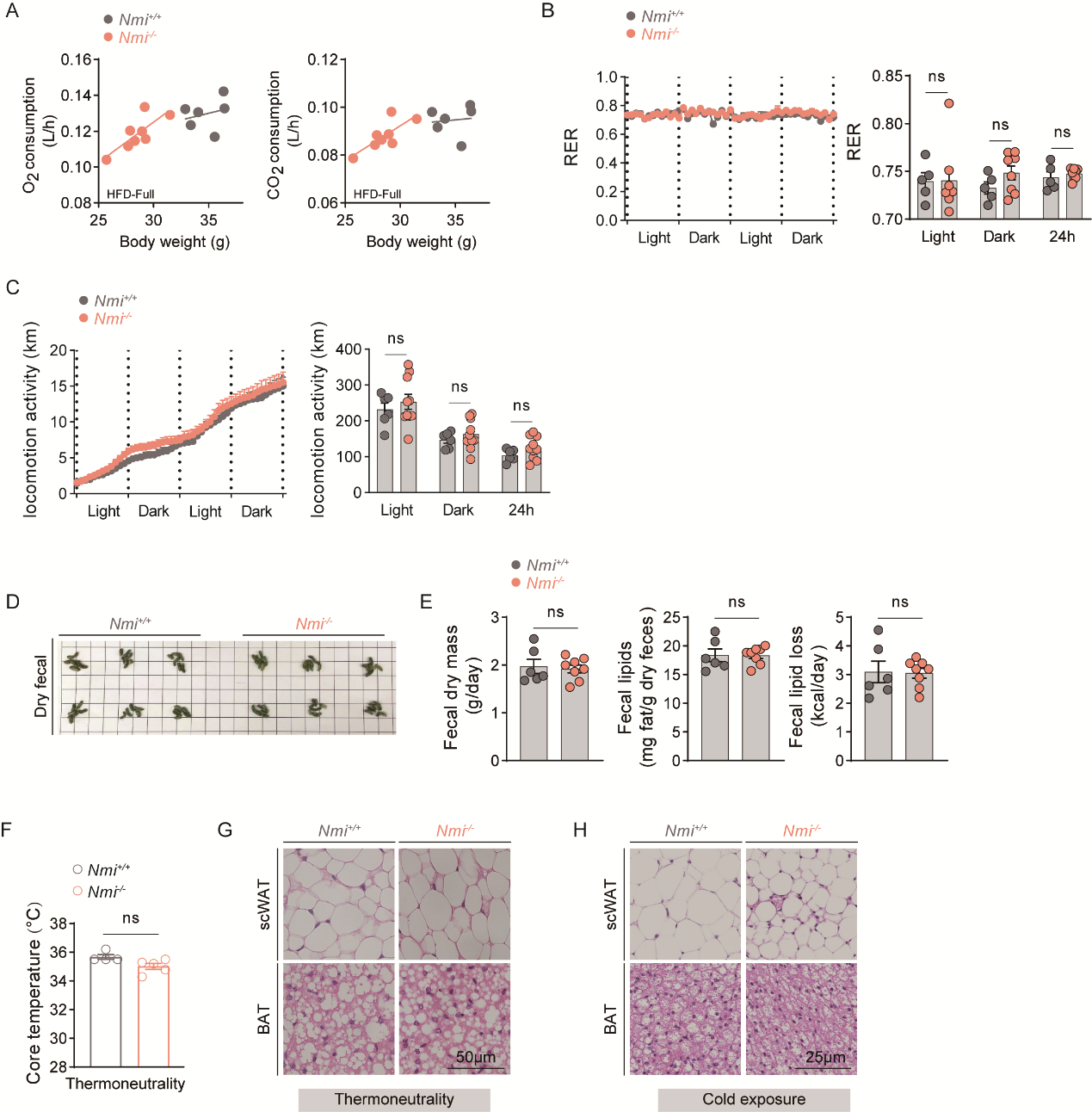


**Figure S3. *Nmi* deficiency does not alter other metabolic parameters under HFD feeding.**

(A) Oxygen consumption (VO_2_) and carbon dioxide production (VCO_2_) in *Nmi^+/+^* and *Nmi^-/-^* mice, analyzed by ANCOVA with body weight (BW) as a covariate (n = 6-8).

(B, C) Respiratory exchange ratio (RER, B) and locomotor activity (C) in *Nmi^+/+^* and *Nmi^-/-^* mice (n = 6-8).

(D) Representative images of feces from *Nmi^+/+^* and *Nmi^-/-^* mice.

(E) Fecal dry mass, lipid content, and calculated fecal lipid loss in *Nmi^+/+^* and *Nmi^-/-^* mice (n = 6-8).

(F) Core body temperature in *Nmi^+/+^* and *Nmi^-/-^* mice at room temperature (n = 4-5).

(G, H) Representative H&E staining of scWAT and BAT under thermoneutral (G) and cold exposure (H) conditions from *Nmi^+/+^* and *Nmi^-/-^* mice.

Data are represented as mean ± SEM. Statistical analyses: two-tailed unpaired Student’s t-test (B, C, E, F). **p* < 0.05, ***p* < 0.01, and ****p* < 0.001; ns, not significant.


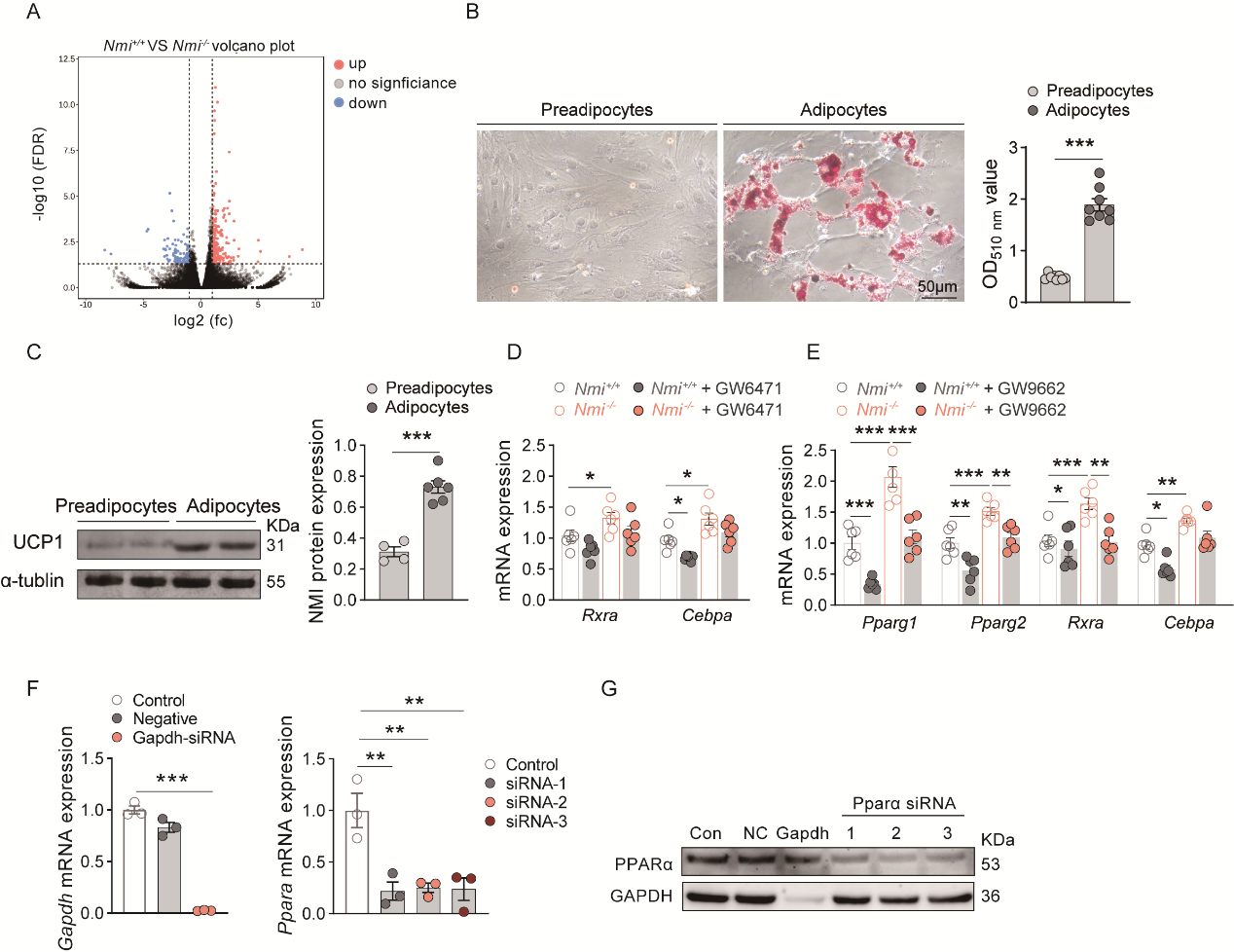


**Figure S4. PPAR signaling is altered in *Nmi*-deficient adipocytes.**

(A) Volcano plot of differentially expressed genes from BAT RNA sequencing (|log2 fold change| > 1, FDR < 0.05) (n = 4).

(B, C) Oil Red O staining (B, n = 8) and UCP1 protein levels (C, n = 4) in preadipocytes (Day 0) and mature adipocytes (Day 8).

(D, E) mRNA expression of PPAR pathway-related genes in mature adipocytes treated with GW6471 (PPARα antagonist, 10 μM) and GW9662 (PPARγ antagonist, 10 μM) for 24 hours (n = 6).

(F, G) mRNA (F) and protein (G) expression of PPAR pathway-related genes in mature adipocytes treated with scramble siRNA and *Ppara* siRNA (100 nM) for 7 days (n = 3).

Data are represented as mean ± SEM. Statistical analyses: two-tailed unpaired Student’s t-test (B, C), one-way ANOVA followed by Bonferroni’s multiple comparisons test (D-F). **p* < 0.05, ***p* < 0.01, and ****p* < 0.001; ns, not significant.


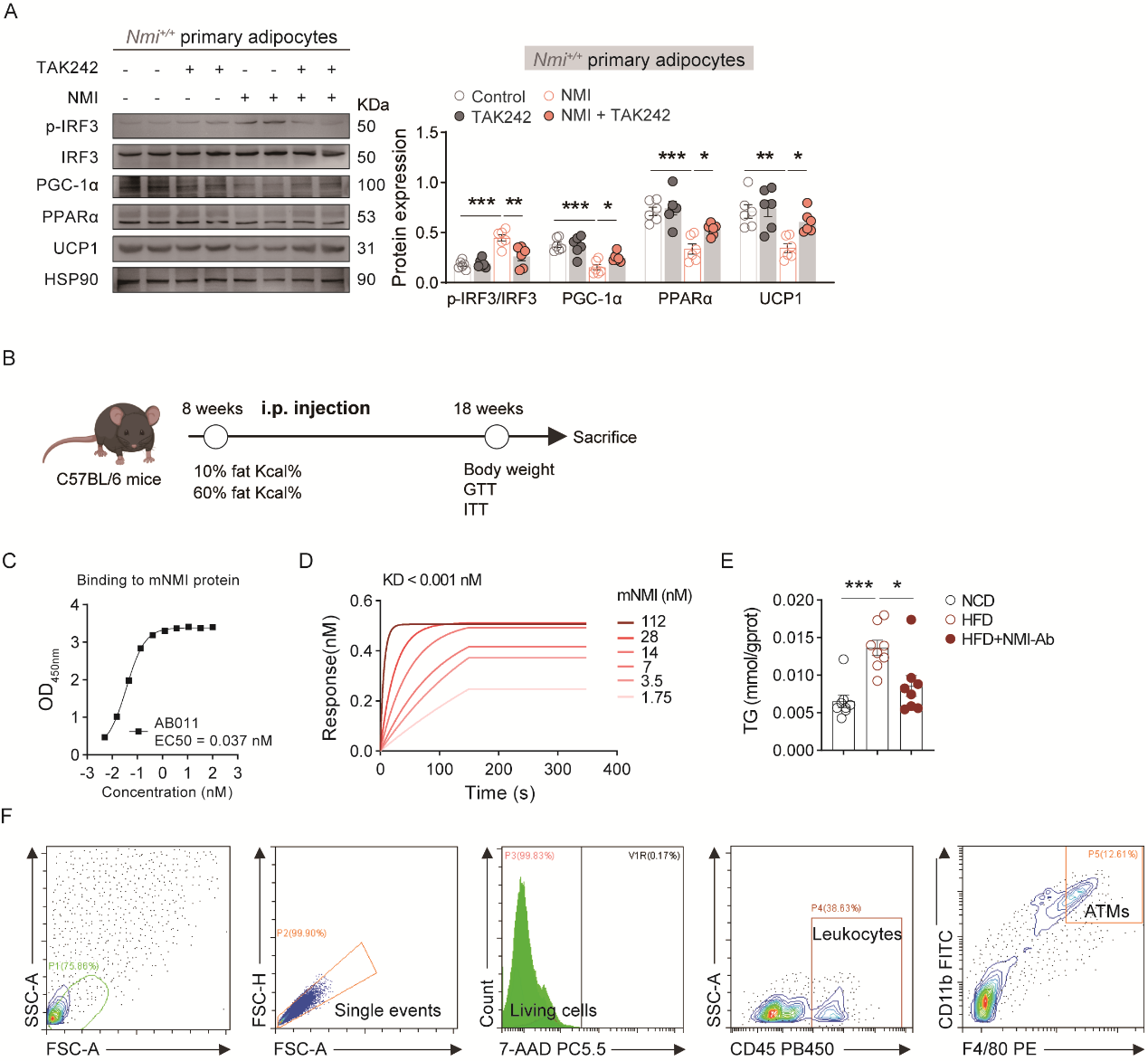


**Figure S5. NMI-neutralizing antibody (mAb-NMI) treatment in DIO mice.**

(A) Mature adipocytes pretreatment with TAK-242 (1 µM, TLR4 inhibitor) for 2 hours, followed by recombinant NMI (5 μg mL^-1^) treatment for 24 hours. Thermogenic proteins are subsequently analyzed by Western blotting (n = 6).

(B) Schematic of mAb-NMI treatment regimen.

(C) Dose-response curve of mAb-NMI binding to recombinant NMI by ELISA. Half-maximal effective concentration (EC₅₀) = 0.037 nM.

(D) Binding kinetics of mAb-NMI to NMI by bio-layer interferometry (BLI).

(E) Hepatic triacylglycerol (TAG) levels in DIO mice treated with mAb-NMI or PBS (n = 8).

(F) Representative flow cytometry gating strategy for WAT macrophages (n = 3-4).

Data are represented as mean ± SEM. Statistical analyses: one-way ANOVA followed by Bonferroni’s multiple comparisons test (A, E). **p* < 0.05, ***p* < 0.01, and ****p* < 0.001, ns, not significant.

**Tables**

Table S1. Primer sequences for *Nmi^+/+^* and *Nmi^-/-^* mice genotyping

| Primer name | Primer sequences (5’to 3’) |
| --- | --- |
| *Nmi* | Forward: CTTAGGGGAGGGAGATTGGC  Reverse: TGGAATTCTCTGGCATCCGA |

Table S2. Primer sequences of mice in Q-PCR

| Mouse genes | Primer sequences (5’to 3’) |
| --- | --- |
| *Rn18s* | Forward: GTAACCCGTTGAACCCCATT  Reverse: CCATCCAATCGGTAGTAGCG |
| *Nmi* | Forward: TGAGGAGCAGACAAGGGAC  Reverse: CAGCAACGCTATGGCACT |
| *Ucp1* | Forward: AGGCTTCCAGTACCATTAGGT  Reverse: CTGAGTGAGGCAAAGCTGATTT |
| *Elovl3* | Forward: TCCGCGTTCTCATGTAGGTCT  Reverse: GGACCTGATGCAACCCTATGA |
| *Dio2* | Forward: AATTATGCCTCGGAGAAGACCG  Reverse: GGCAGTTGCCTAGTGAAAGGT |
| *Prdm16* | Forward: GACATTCCAATCCCACCAGA  Reverse: CACCTCTGTATCCGTCAGCA |
| *Acoxl* | Forward: TAACTTCCTCACTCGAAGCCA  Reverse: AGTTCCATGACCCATCTCTGTC |
| *Cox5b* | Forward: ACCCTAATCTAGTCCCGTCC  Reverse: CAGCCAAAACCAGATGACAG |
| *Cpt1a* | Forward: CTCCGCCTGAGCCATGAAG  Reverse: CACCAGTGATGATGCCATTCT |
| *Cpt1b* | Forward: GCACACCAGGCAGTAGCTTT  Reverse: CAGGAGTTGATTCCAGACAGGTA |
| *Cpt2* | Forward: CAGCACAGCATCGTACCCA  Reverse: TCCCAATGCCGTTCTCAAAAT |
| *Ppargc1a* | Forward: TATGGAGTGACATAGAGTGTGCT  Reverse: CCACTTCAATCCACCCAGAAAG |
| *Tfam* | Forward: CTGTTCCGGGGAATGTGGAG  Reverse: CTGATAGACGAGGGGATGCG |
| *Nrf1* | Forward: AATGACCCAGGCTCAGCTTC  Reverse: GCTTGCAGCTTTCTTTCCCC |
| *Sdhb* | Forward: AATTTGCCATTTACCGATGGGA  Reverse: AGCATCCAACACCATAGGTCC |
| *Ppara* | Forward: GCGTACGGCAATGGCTTTAT  Reverse: GAACGGCTTCCTCAGGTTCTT |
| *Pparg* | Forward: TGGCATCTCTGTGTCAACCAT  Reverse: GGCATGGTGCCTTCGCTGA |
| *Pparg1* | Forward: GGACTGTGTGACAGACAAGATTTGA  Reverse: CTGAATATCAGTGGTTCACCGC |
| *Pparg2* | Forward: CTCTGTTTTATGCTGTTATGGGTGA  Reverse: GGTCAACAGGAGAATCTCCCAG |
| *Rxra* | Forward: ATGGACACCAAACATTTCCTGC  Reverse: CCAGTGGAGAGCCGATTCC |
| *Cebpa* | Forward: CAAGAACAGCAACGAGTACCG  Reverse: GTCACTGGTCAACTCCAGCAC |
| *Cebpb* | Forward: GCGCGAGCGCAACAACATC  Reverse: TGCTTGAACAAGTTCCGCAG |
| *Gapdh* | Forward: TTCACCACCATGGAGAAGGC  Reverse: GGCATGGACTGTGGTCATGA |
| *Il6* | Forward: TGCTGGTGACAACCACGGCC |
|  | Reverse: GTACTCCAGAAGACCAGAGG |
| *Il1b* | Forward: CAACCAACAAGTGATATTCTCCATG |
|  | Reverse: GATCCACACTCTCCAGCTGCA |
| *Tnf* | Forward: CCTGTAGCCCACGTCGTAG |
|  | Reverse: GGGAGTAGACAAGGTACAACCC |

Table S3. List of siRNA sequence

| siRNA sequence name | siRNA sequences (5’to 3’) |
| --- | --- |
| Negative control siRNA | UUCUCCGAACGUGUCACGUTT |
|  | ACGUGACACGUUCGGAGAATT |
| *Gapdh* siRNA | UCACUCAAGAUUGUCAGCAAUGCAU AUGCAUUGCUGACAAUCUUGAGUGA |
| *Ppara* siRNA-1 | GCUAGUGUCCGAUAGACAATT |
|  | UUGUCUAU CGGACACUAGCTT |
| *Ppara* siRNA-2 | GGUUAAGAAUAUUAGAGUATT |
|  | UACUCUAA UAUUCUUAACCTT |
| *Ppara* siRNA-3 | GGCUGUAACUUAAUCUUAATT |
|  | UUAAGAUU AAGUUACAGCCTT |
